# A case of aortic dissection in a cocaine abuser: a case report and review of literature

**DOI:** 10.1186/1757-1626-1-369

**Published:** 2008-12-02

**Authors:** Maen Nusair, Jamil Y Abuzetun, Azamuddin Khaja, Mary Dohrmann

**Affiliations:** 1 Department of Internal Medicine, University Missouri Columbia, 1-Hospital Dr. Columbia, MO 65212-0001, USA; 2 Department of Internal Medicine, Creighton University Medical Center, Omaha, NE 68198, USA; 3 Division of cardiovascular medicine, University of Iowa, Iowa City, IA 52242-1009, USA; 4 Division of cardiovascular medicine, University of Missouri-Columbia, 1-Hospital Dr. Columbia, MO 65212-0001, USA

## Abstract

**Background:**

Prompt diagnosis and management of aortic dissection are key to reduce patient morbidity and mortality; hence the need to a have a high index of suspicion for this condition. We believe it's important to report this case because it underscores the relationship between cocaine abuse and aortic dissection. In addition it strongly emphasizes basic principles in medicine: patients should not be profiled, and chronic complaints may need reassessment.

**Case Presentation:**

We are presenting a case of Stanford type A aortic dissection in a 46 year old patient with history of cocaine abuse. The aortic dissection presented as worsening of chronic upper abdominal pains he has had for years. He presented to us hours after using crack cocaine.

**Conclusion:**

Aortic dissection associated with cocaine abuse develops at a younger age. Therefore it's crucial to have high index of suspicion for aortic dissection in this subset of patients. Furthermore as this case illustrates, serious diseases can masquerade in old complaints. Patients should never be profiled, and chronic complaints should always be revisited.

## Background

Aortic dissection is the most serious condition of the human aorta. Prompt diagnosis and management are key to reduce patient morbidity and mortality; hence the need to a have a high index of suspicion for this condition. The association between cocaine abuse and aortic dissection is well established in literature, and is seen in approximately 1% of patients. According to a 2005 National Survey on Drug Use and Health approximately 33.7 million Americans ages 12 and older had tried cocaine at least once in their lifetimes, representing 13.8% of that population. We present a patient with history of chronic abdominal pain. He presented to our emergency department with mild worsening of that pain hours after using crack cocaine and was found to have a dissecting aortic aneurysm.

This case is a strong reminder of the association between a major public health problem and a grave life threatening disease. In addition, this case emphasizes basic principles in medicine: patients should not be profiled, and chronic complaints may need reassessment.

## Case Presentation

The patient is a 46-year-old male with a past medical history of chronic pancreatitis and chronic upper abdominal pain, hypertension, alcoholism, and cocaine abuse. He arrived at the emergency department (ED) a few hours after he developed sudden, severe worsening of his epigastric pain, which the patient described as similar to his previous chronic pancreatitis associated pain flare-ups. The pain was stabbing, radiating to the back and was associated with nausea and profuse sweating. Shortly after arrival to the ED, he complained of pain extending to his chest.

On physical examination his blood pressure was 150/80 and pulse 68 beats per minute. He had tenderness to palpation over his upper abdomen, especially in the epigastrium with associated guarding. The chest, lung, and cardiac exams were reported as normal; the remainder of the physical examination was unremarkable.

Lab results were all within normal limits. Electrocardiogram showed sinus tachycardia but no acute ischemic changes. Although the impression was that the pain was most likely attributable to his chronic pancreatitis, an abdominal CT with intravenous contrast was performed to rule out other possible etiologies for his acute abdominal pain.

**Figure 1:**
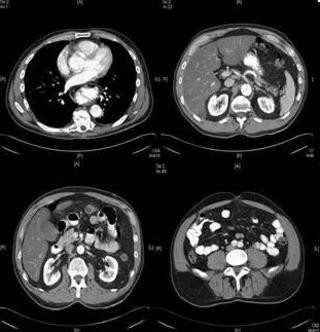
CT scans done on admission day; notice the aortic intimal flap in ascending aorta, descending thoracic aorta, abdominal aorta and common iliac arteries.

The abdominal CT revealed an aortic dissection extending from the upper abdomen to the level of the common iliac arteries. A chest CT verified the origin of the dissection at the aortic root [Figure 1]. The patient was started on intravenous Esmolol and nitroprusside. The cardiothoracic surgery team was consulted, and the patient was taken to the operating room emergently.

**Figure 2:**
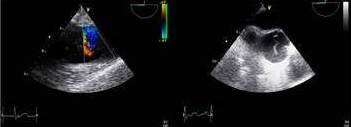
Transesophageal echocardiogram revealing intimal flap in ascending aorta.

A transesophageal echocardiogram [Figure 2] performed in the operating room revealed dilation of the aortic root, an intimal flap, and aortic regurgitation. During the thoracotomy an aortic root hematoma was noted in addition to a right coronary artery dissection with blood oozing into the pericardial space. The patient underwent a replacement of both the ascending aorta and the aortic valve, followed by over sewing of the proximal right coronary at the site of the dissection and bypass grafting with a saphenous venous graft.

The patient did well after the surgery and we were able to take more medical history. We found out that hours prior to development of the above symptoms the patient smoked crack cocaine.

The patient continued to do well and it was decided to treat the dissection of abdominal aorta medically. He was discharged home on a beta blocker with close follow up arranged.

## Discussion

Aortic dissection is the most lethal disease of the aorta. Its incidence is estimated to be 3 in a 1000 cases according to the international registry of aortic dissection (IRAD). If left untreated 33% of individuals will die within 24 hours of presentation, and 50% die in the initial 48 hours [1]. In 90–95% of cases an intimal tear is the primary event that initiates aortic dissection. Blood dissects through the tear, and a mural hematoma starts developing longitudinally, circumferentially, or both, forming a false lumen [2]. It is postulated that thinning of the media due to degeneration of collagen, elastin or muscles within the media (cystic medial degeneration), weakens the intima and predisposes it to tearing. This degeneration may be related to conditions like Marfan syndrome, Ehlers-Danlos disease and other connective tissue disease. This degenerative process might also be produced by obstruction of the vaso vasora by an atherosclerotic plaque. The second mechanism proposed to explain aortic dissection is the rupture of a vaso vasorum within the tunica media, which then dissects and ruptures through the intima [1]. This type of dissection occurs in 5–10% of cases.

About 90% of aortic dissections occur in the proximal 10 cm of the aortic root; the remaining 10% originate distal to the left subclavian artery [1]. The Stanford classification and DeBakey's classification are used in aortic dissection. Stanford classification is more widely used, it classifies dissection into: Type A which involves the ascending aorta, and type B which is any dissection that doesn't involve the ascending aorta.

**Figure 3:**
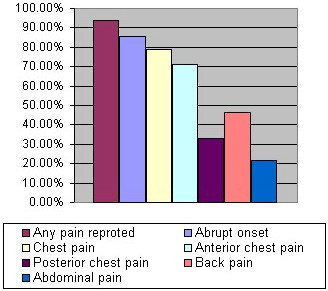
Different characteristics of pain associated with Stanford type A dissection.

According to an IRAD review, the most common presenting complaint was pain. This pain is typically abrupt and intense from the onset. The site of the pain is often indicative of the site of dissection. Anterior chest pain is typical in ascending aorta dissection; neck and jaw pain may indicate dissection involving the arch and carotid arteries. Interscapular tearing pain suggests dissection of the descending aorta. [Figure 3 demonstrates the different pain characteristics associated Stanford types A dissection].

Other symptoms are attributed to decreased organ perfusion. Focal neurological symptoms, including limb paresthesia and weakness, indicate involvement of spinal arteries. Evidence of limb ischemia or mesenteric ischemia suggests compromise of their respective arterial supplies. Symptoms can also be caused by organ compression by an expanding hematoma. Dyspnea suggests tracheal or bronchus compression, dysphagia results from esophageal compression, and hoarseness indicates recurrent laryngeal nerve compression. A superior vena caval syndrome may be precipitated by compression of the superior vena cava.

Physical examination may reveal hypertension or hypotension, the latter being an ominous sign. Hypotension may be a sign of critical complications (e.g. cardiac tamponade, rupture of the aorta), but it can also be due to hypervagotonia. Pulse deficit (defined as weak or absent pulse in the carotid, brachial or femoral arteries) is more common in acute Stanford type A dissection compared to type B (19% versus 9% respectively) [3]. Pulse deficit is identified in 15% of cases and is generally associated with greater complications and mortality rates. A disparity of greater than 20 mm Hg in the systolic blood pressure of each arm also raises suspicion for aortic dissection.

A chest x-ray is typically the first imaging modality used. Typical abnormalities include a widened mediastinum (seen in 25% of cases) and abnormal aortic contour.

Other findings include: tracheal deviation, esophageal deviation, depression of the left main bronchus and left apical cap. In the IRAD review 12% of initial chest x-rays were reported as normal [3].

Aortography was previously considered the gold standard test for diagnosis. Aortography identifies the false and true lumens, assesses involvement of the arch vessels, and detects aortic valve insufficiency. However, aortography is also an invasive, time-consuming technique that requires the use of potentially nephrotoxic contrast. The European cooperative study demonstrated that the diagnostic accuracy of aortography is not as high as originally thought, with sensitivity of 88% and specificity of 94%. Under-diagnosis of aortic dissection with aortography can be due to thrombosis of the false lumen or the simultaneous opacification of both the false and true lumens. Noninvasive imaging modalities, such as spiral CT, multiplanar transesophageal echocardiogram (TEE), and magnetic resonance imaging (MRI) are replacing aortography for purpose of evaluating for aortic dissection. The diagnostic accuracies of these studies for the diagnosis of Stanford type A dissection vary from one study to another; however, most studies agree that their sensitivities and specificities are comparable [see Table 1 below]. TEE has the advantage that patients don't need to be transported for the procedure; it doesn't require the use of potentially nephrotoxic contrast agents and provides functional evaluation of aortic valve.

**Table 1:** Comparison between imaging modality used for diagnosis of Type A aortic dissection.

**Imaging technique**	**Sensitivity**	**Specificity**
TEE	98 (95–99)	95 (92–97)

Helical CT	100 (96–100)	98 (87–99)

MRI	98 (95–99)	98 (95–100)

Initial management of aortic dissection includes control of blood pressure and heart rate to decrease the shear forces on the dissected aorta. Intravenous beta blockers (e.g. labetalol or metoprolol) are the mainstay of medical treatment. If needed, vasodilatation with agents such as sodium nitroprusside or intravenous calcium channel blockers can be used. The goal of surgical treatment with type A aortic dissection is to alleviate symptoms, control complications and prevent aortic rupture.

The procedure entails removing the intimal flap, obliterating the false lumen and restoring the continuity of the aorta.

The development of the aortic dissection in our patient may have been associated with cocaine use. Histopathology of the resected aorta was normal; the fact he smoked crack cocaine hours prior to admission implicates an association. Although association between cocaine and aortic dissection is well established, cocaine seems to be associated with less than 1% of all aortic dissections [4]. In a study by Hsue and colleagues of 40 patients with aortic dissection presenting to an inner city hospital, 37% of patients reported cocaine use minutes to hours prior to presentation. The study concluded that young age, black race and hypertension are risk factors associated with aortic dissection in cocaine users [5].

The proposed mechanism to explain the association between cocaine use and dissection suggests that the catecholamine surge associated with cocaine use and the subsequent elevation of blood pressure and heart rate increase shear forces on the intima and may cause it to tear. Given the small percentage of patients who develop aortic dissection compared to the large number of cocaine abusers, it is postulated that those who develop dissection have an underlying aortopathy. In Hsue and colleagues report, 79% of patients were hypertensive; it is possible that the theorized underlying aortopathy in their population of patients is hypertension-related. Chronic cocaine also accelerates atherosclerosis [6]; therefore it is plausible that patients have an atherosclerotic aortopathy predisposing them to aortic dissection, albeit such evidence was lacking in the patient reported in the present case report.

## Prognosis

According to IRAD the in-hospital mortality for promptly surgically treated patients was 26.9% compared to 56.2% for those treated only medically. A study based on IRAD data suggests that for patients treated medically, long-term, ongoing medical treatment may provide some benefit [7]. Long-term survival was influenced most by preexisting risk factors e.g. history of atherosclerosis and previous cardiac surgery.

## Conclusion

Aortic dissection is an uncommon but potentially fatal condition. Reliable modalities of diagnosis are available but high index of suspicion remains key for prompt diagnosis and management. Cocaine abuse is an established precipitator of aortic dissection, although it is a rare, occurring in less than 1% of patients. Young age, black race and hypertension are risk factors associated with aortic dissection in cocaine users, two of which were present in our patient. Because of the small number of patients identified with cocaine-associated aortic dissection, more still needs to be known about the other possible risk factors and underlying pathophysiology.

This case reminds us how serious diseases can masquerade in old complaints. Patients should never be profiled and chronic complaints should always be revisited.

## Abbreviations

IRAD: International Registry of Acute Aortic Dissection; ER: Emergency Department; CT: Computed Tomography; TEE: Transesophageal Echocardiography; MRI: Magnetic Resonance Imaging.

## Consent

Written informed consent was obtained from next of kin for publication of this case report and accompanying images. A copy of the written consent is available for review by the Editor-in-Chief of this journal. The patient has died from causes unrelated to our case.

## Competing interests

The authors declare that they have no competing interests.

## Authors' contributions

MN interacted with patient, reviewed literature and wrote manuscript, JYA and AK considerably contributed to writing the manuscript, MD critically reviewed and modified manuscript.

All authors read and approved the final manuscript.
